# Non-Canonical Activation of the Epidermal Growth Factor Receptor by Carbon Nanoparticles

**DOI:** 10.3390/nano8040267

**Published:** 2018-04-23

**Authors:** Daniel Stöckmann, Tim Spannbrucker, Niloofar Ale-Agha, Philipp Jakobs, Christine Goy, Nadine Dyballa-Rukes, Tamara Hornstein, Alexander Kümper, Annette Kraegeloh, Judith Haendeler, Klaus Unfried

**Affiliations:** 1 IUF—Leibniz-Institut für Umweltmedizinische Forschung, Auf’m Hennekamp 50, 40225 Düsseldorf, Germany; daniel_stoeckmann@email.de (D.S.); Tim.Spannbrucker@IUF-duesseldorf.de (T.S.); Niloofar.ALE-AGHA@uni-duesseldorf.de (N.A.-A.); Philipp.Jakobs@IUF-duesseldorf.de (P.J.); Christine.Goy@IUF-Duesseldorf.de (C.G.); Nadine.Dyballa@uni-duesseldorf.de (N.D.-R.); Tamara.Hornstein@IUF-duesseldorf.de (T.H.); juhae001@uni-duesseldorf.de (J.H.); 2 INM—Leibniz-Institut für Neue Materialien, Campus D2 2, 66123 Saarbrücken, Germany; akuempi@web.de (A.Kü.); Annette.Kraegeloh@leibniz-inm.de (A.Kr.); 3 Medizinische Fakultät, Heinrich-Heine-Universität Düsseldorf, 40225 Düsseldorf, Germany

**Keywords:** tyrosine kinase receptor, caveolin-1, airway epithelium, lung inflammation, protein kinase B

## Abstract

The epidermal growth factor receptor (EGFR) is an abundant membrane protein, which is essential for regulating many cellular processes including cell proliferation. In our earlier studies, we observed an activation of the EGFR and subsequent signaling events after the exposure of epithelial cells to carbon nanoparticles. In the current study, we describe molecular mechanisms that allow for discriminating carbon nanoparticle-specific from ligand-dependent receptor activation. Caveolin-1 is a key player that co-localizes with the EGFR upon receptor activation by carbon nanoparticles. This specific process mediated by nanoparticle-induced reactive oxygen species and the accumulation of ceramides in the plasma membrane is not triggered when cells are exposed to non-nano carbon particles or the physiological ligand EGF. The role of caveolae formation was demonstrated by the induction of higher order structures of caveolin-1 and by the inhibition of caveolae formation. Using an in vivo model with genetically modified mice lacking caveolin-1, it was possible to demonstrate that carbon nanoparticles in vivo trigger EGFR downstream signaling cascades via caveolin-1. The identified molecular mechanisms are, therefore, of toxicological relevance for inhaled nanoparticles. However, nanoparticles that are intentionally applied to humans might cause side effects depending on this phenomenon.

## 1. Introduction

The epidermal growth factor receptor (EGFR) is an omnipresent receptor tyrosine kinase, which can be activated by the binding of specific ligands. It triggers intracellular signaling pathways involved in a plethora of cellular responses to external stimuli including proliferation, apoptosis, and pro-inflammatory reactions. As a functional protein located in the plasma membrane, the EGFR might be affected when cells are intentionally or unintentionally exposed to nanoparticles. Important evidence for an interference of nanoparticles with EGFR signaling comes from toxicological approaches using different kinds of nanoparticles in various experimental systems. Colloidal nanoparticles consisting of gold, silver, or iron oxide were demonstrated to induce changes in EGFR-dependent signaling and the expression of gene products regulated by this signaling network in a human epithelial cell line [1]. Earlier investigations of lung epithelial cells exposed to pure hydrophobic carbon nanoparticles demonstrated that a proliferative response on this kind of exposure is mediated by the activation of the EGFR [2,3]. These reactions proved to be specific for nanoparticles since non-nano carbon particles failed to induce these reactions.

The molecular mechanism by which nanoparticles interfere with EGFR-signaling are of particular importance for identifying hazards of nanoparticles and for developing safe nanomaterials [4]. EGFR might be activated by natural ligands released by nanoparticle-triggered cell reactions. Evidence for this kind of mechanism comes from studies with an epithelial cell line exposed to different kinds of environmentally relevant particles and amorphous silica nanoparticles [5,6]. The exposure to these xenobiotics appears to activate TACE (tumour necrosis factor-α-converting enzyme), which is able to shed the ectodomain of TGF-α known as a ligand of EGFR. Signaling events, which are crucial for the pro-inflammatory response in these cells, appears to be activated by this pathway of specific ligand binding. However, the receptor might also be activated by rather unspecific cellular stressors, which are described for oxygen radicals [7]. In our earlier studies, we demonstrated that carbon nanoparticles are able to induce oxidative stress in different kinds of cells [8,9]. The intrinsic oxidative capacity of carbon nanoparticles leads to a rapid generation of reactive oxygen species [10]. In this context, we showed that intracellular reactive oxygen species are crucial for activating proliferative EGFR signaling in lung epithelial cells exposed to carbon nanoparticles [9,10]. Involving membrane-linked src-family kinases (SFK) downstream of EGFR, the activation of protein kinase B (Akt) and proliferative and pro-inflammatory mitogen-activated protein kinase (MAPK) signaling pathways were specifically triggered [11,12]. Molecular analyses of the lipid composition of exposed cells demonstrated that exposure to carbon nanoparticles led to an increase of ceramides in lipid raft membrane fractions, which caused the internalization and activation of the EGFR [9]. Interestingly, ceramide-induced receptor activation could be prevented by stabilizing the EGFR in lipid raft fractions of exposed cells. This effect was achieved by adding ectoine, which is an extremolyte known to stabilize the interaction of membrane proteins with the lipid bilayer [13]. Using this intervention strategy, we were able to demonstrate that the pro-inflammatory effects of nanoparticle-induced EGFR signaling are relevant to in vivo experiments in rats and mice [14].

Based on these previous findings, we now ask whether EGFR activation triggered by carbon nanoparticles involves specific non-canonical mechanisms, which can be distinguished from ligand-dependent activation. As Filosto et al. [15] have shown, non-canonical activation of the EGFR by reactive oxygen species is characterized by src family kinase- (SFK-) dependent processes including the generation of ceramides. After canonical ligand binding, no EGFR homodimers are formed. Moreover, activated monomers are internalized by the formation of caveolae and transported to the perinuclear region where they remain relatively stable compared to ligand-activated homodimers which are, after clathrin-dependent internalization, rapidly subjected to lysosomal degradation [16,17]. In this context caveolin-1, a protein involved in many regulatory processes is of particular importance. The oligomerization of caveolin-1 is the main structural event for the formation of caveolae, which is a specific form of endocytotic membrane invaginations. The lipid composition of the plasma membrane appears to have an impact on EGFR activation mediated by caveolin-1. Ganglioside GM3 has been identified as a negative regulator of EGFR by modulating caveolin-1 levels in raft and non-raft regions of the plasma membrane [18]. In our own studies, we observed a rapid dramatic loss of GM3 accompanied by an accumulation of ceramides after exposure of lung epithelial cells to carbon nanoparticles [9].

The current study aimed to identify signaling events triggered by carbon nanoparticles interacting with epithelial cells. Nanoparticle-specific activation of EGFR was investigated in the model system of the lung epithelium. Using an alveolar type II-derived epithelial cell line (RLE-6TN), we aimed to discriminate non-canonical events from ligand-dependent receptor activation. As a possible mediator of non-canonical EGFR activation, the role of caveolin-1 in lung epithelial cells in vitro and in vivo was investigated. By comparing molecular events triggered by carbon nanoparticles and the natural ligand of EGFR, the epidermal growth factor (EGF), which is the importance of non-canonical EGFR activation, was elucidated. Employing different kinds of intervention strategies including pharmacological inhibitors also knock out animals for caveolin-1. The relevance of these molecular events was documented.

## 2. Results

### 2.1. Particle Characterization

Particles used in this study consist of aciniform, which is an elemental carbon. Carbon nanoparticles (CNP) as well as (non-nano) carbon particles (CP) were characterized for physical properties by using transmission electron microscopy and dynamic light scattering, which was described in the material and methods section and in an earlier publication [19]. Results are shown in Figure S1 and Table S1 in Supplementary Materials.

### 2.2. Caveolin-1 Is Involved in EGFR Activation after Carbon Nanoparticle Exposure

**Figure 1:**
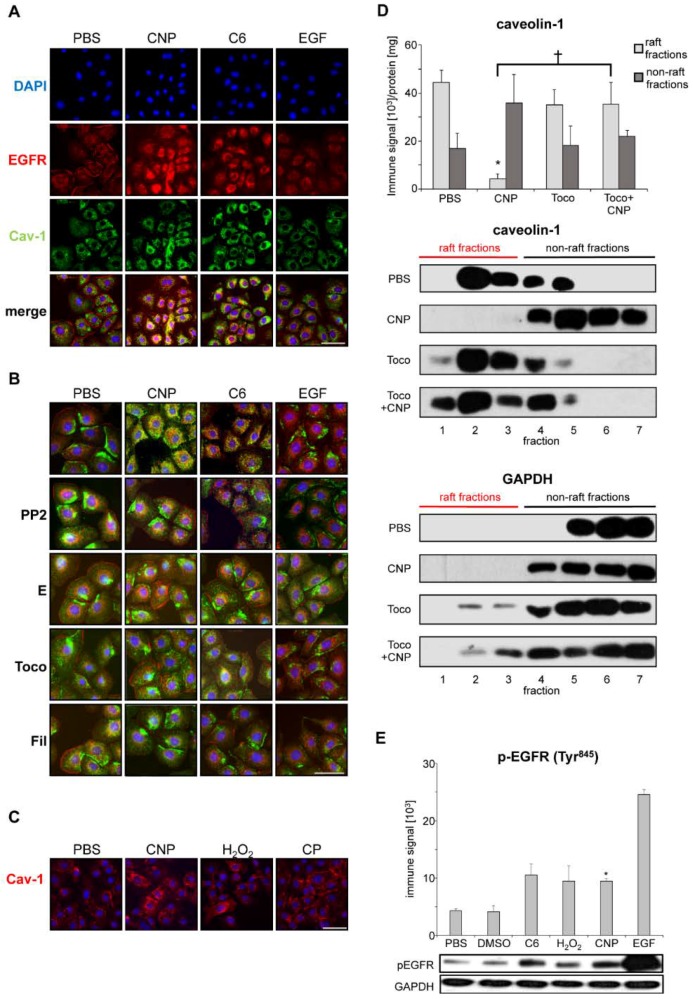
Caveolin-1 and EGFR co-localization as a feature of non-canonical EGFR activation. Epithelial cells (RLE-6TN) were exposed (5 min) to carbon nanoparticles (CNP), non-nano carbon particles (CP), each 10 µg/cm^2^, (50 µM) H_2_O_2_, (5 µM) C6 ceramide, or EGF (100 ng/mL), respectively. (**A**) Subcellular localization of EGFR (red Alexa flour 594) and caveolin-1 (green Alexa flour 488). Co-localization is visualized by the yellow color in merged images; (**B**) Subcellular localization of EGFR and caveolin-1 in cells pre-treated with inhibitors of carbon nanoparticle-specific signaling prior to particle or EGF exposure: SFK inhibitor PP2 (10 µM), 1 mM ectoine (**E**), 75 µM α-tocopherol (Toco), and 1 µg/mL filipin III (Fil). Co-localization is visualized by the yellow color in merged images; (**C**) Subcellular localization of caveolin-1 (red Alexa fluor 594) after exposure to carbon nanoparticles (CNP), carbon particles (CP), or hydrogen peroxide (H_2_O_2_); (**D**) Quantification and representative Western blots of caveolin-1 in lipid raft fraction of RLE-6TN cells exposed to CNP (10 µg/cm^2^). Raft and non-raft fraction were isolated from density gradients after ultracentrifugation. Pre-treatment of cells with α-tocopherol (Toco) was applied as an antioxidant strategy. GAPDH was used as a control protein not associated with lipid rafts. The bars in the graph represent the additive immune signals of raft and non-raft fractions, which was indicated in the representative original Western blots; (**E**) Quantification and representative Western-blot of EGFR phosphorylation at Tyr^845^. Nuclei were stained with DAPI (blue). Scale bars represent 20 µm; *, which was significantly different to PBS control (*p* < 0.05); †, significantly different from CNP alone (*p* < 0.05).

In the first set of experiments, we aimed to identify signaling events, which allow us to discriminate non-canonical EGFR activation by carbon nanoparticles from ligand-dependent activation. Therefore, the role of caveolin-1 as a potential mediator of these events was investigated in a well characterized rat lung epithelial cell line [20]. Analyses of subcellular localization of EGFR and caveolin-1 were performed by fluorescence microscopy with specific antibodies. Cells either exposed to suspensions of carbon nanoparticles (10 µg/cm^2^) or to EGF as the natural ligand were compared. Earlier, we demonstrated that ceramides are accumulated in the cell membrane after exposure to carbon nanoparticles. Therefore, ceramide (C6) was applied in order to evaluate the role of this nanoparticle-specific event for non-canonical EGFR activation [9]. After five minutes of exposure, all three stimuli led to a translocation of the EGFR from the plasma membrane into the cytoplasm, which is considered a feature of receptor activation (see Figure 1A). Simultaneously, intracellular caveolin-1 accumulation occurred after exposure to carbon nanoparticles and ceramide but not after EGF application. The co-localization of EGFR and caveolin-1 in the cytoplasm of the cells is visualized by the yellow signals in the merge of the images. The co-localization of EGFR and caveolin-1 was only observed after particle and ceramide exposure but not in the presence of the natural ligand EGF. Therefore, the intracellular accumulation of caveolin-1 and its co-translocation with the EGFR can be considered a specific feature that allows to discriminate ligand-dependent from non-canonical receptor activation.

In order to verify the specificity of the observed reactions, a number of molecular events identified earlier to be involved in nanoparticle-specific activation of signaling pathways were investigated by using intervention approaches. Src family kinases (SFK) were inhibited by the pharmacological inhibitor PP2, which specifically and dose dependently diminish downstream signaling [12]. The preventive application of 1 mM ectoine is considered to stabilize EGFR membrane interaction and inhibit the activation and internalization of the receptor. The influence of reactive oxygen species was counteracted by pre-treating the cells with α-tocopherol. Filipin III was used a cholesterol-depleting substance, which is known to prevent the formation of caveolae. All these intervention approaches reduced the internalization and co-localization of EGFR and caveolin-1 after carbon nanoparticle exposure (see Figure 1B). EGF-dependent receptor activation was not influenced by these interventions. From these data points, we can conclude that formation of reactive oxygen species, activation of SFK and structural changes of the membrane including the formation of caveolae are essential components of non-canonical EGFR activation by carbon nanoparticles.

The specificity of the translocation of caveolin-1 from the plasma membrane to the cytoplasm induced by carbon nanoparticles was tested by applying particulate and non-particulate control substances (see Figure 1C). The relevance of reactive oxygen species for signal induction was demonstrated by the translocation of caveolin-1 in cells exposed to hydrogen peroxide (50 µM). Non-nano carbon particles (CP) with a primary size of >200 nm (see supplementary files for particle characterization) were not able to trigger the specific signaling events. In our earlier studies, we were able to show that these particles at equal mass doses are not able to trigger EGFR translocation and activation as well as subsequent signaling steps and endpoints [3,9,12]. Dose response experiments suggest that this effect is linked to the considerably reduced surface area of the bigger particles compared to the nanoparticles of equal mass.

The activation of the EGFR by carbon nanoparticles is associated with the shift of the receptor molecule from detergent-resistant lipid rafts to non-raft membrane compartments that can be separated by density centrifugation [21]. We investigated whether caveolin-1 behaves similarly to EGFR in these kind of analyses. Western-blot analyses of density gradient fractions reveal that, after exposure to carbon nanoparticles caveolin-1, EGFR is shifted from the raft to the non-raft fractions (see Figure 1D). This effect could be prevented by the pre-treatment of the cells with the antioxidant α-tocopherol.

As proof of principle that co-localization and translocation of both proteins reflect receptor activation, the activating phosphorylation of the EGFR under the chosen experimental conditions has to be demonstrated. In earlier studies, we demonstrated that Tyr^1173^ phosphorylation as a marker of EGFR autophosphorylation is triggered by carbon nanoparticles through reactive oxygen species and ceramide accumulation [9]. We now tested the phosphorylation status of the receptor at Tyr^845^. This SFK-dependent phosphorylation has been described to be crucial for kinase activity of the EGFR [22,23,24]. Figure 1E demonstrates that conditions under which intracellular co-localization of caveolin-1 and EGFR occurs, the amount of EGFR that is phosphorylated at Tyr^845^ is significantly increased. A similar reaction was observed when EGF was applied as a positive control.

### 2.3. Carbon Nanoparticles Induce Higher Order Structures of Caveolin-1

**Figure 2:**
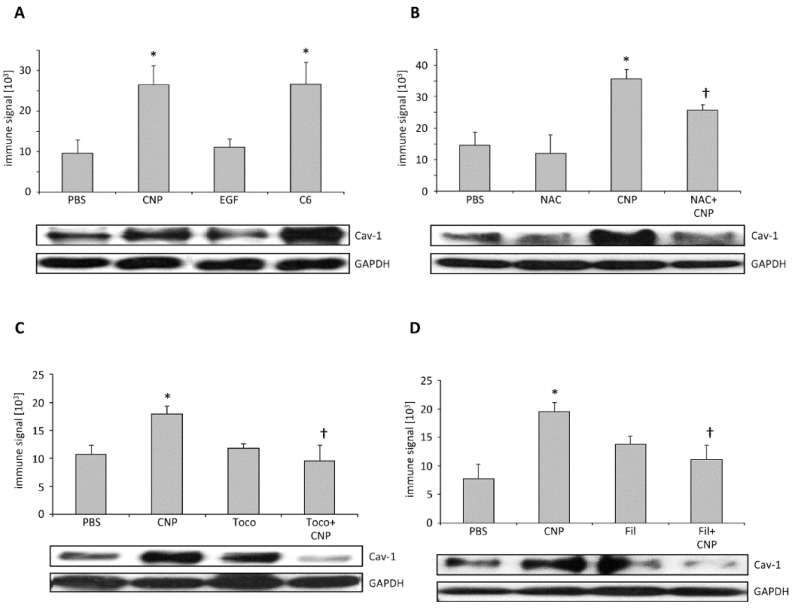
Caveolin-1 protein complexes in dithiobis-succimidylpropionate (DSP)-cross-linked protein extracts. After exposure RLE-6TN cells were treated with DSP (1 mM, 1 h at 4 °C) to stabilize higher order caveolin-1 structures to be detectable by Western blotting. Means and standard errors as well as representative Western-blots are depicted. (**A**) Cells were exposed (5 min) to CNP (10 μg/cm^2^), EGF (100 ng/mL), or C6 ceramide (5 μM). Cell were pre-treated with (18 h) *N*-acetylcysteine (NAC, 1 mM); (**B**), or 1 h with α-tocopherol (Toco, 75 µM) (**C**), or filipin III (Fil, 1 µg/mL) (**D**). *, significantly different to PBS control (*p* < 0.05). †, significantly different from CNP alone (*p* < 0.05).

The formation of caveolae is accomplished by structural organization of caveolin molecules [25]. Oligomerization of caveolin-1 as well as its interaction with other structural proteins like cavins is an essential pre-requisite of caveolar invaginations [26]. As the application of filipin III inhibited EGFR translocation after carbon nanoparticle exposure (see Figure 1B), the formation of caveolae might be a critical step in non-canonical EGFR activation. Protein interactions can be observed by crosslinking proteins in intact cells, by applying the membrane permeable substance DSP (dithiobis-succimidylpropionate), and through subsequent protein analysis [27]. In order to test whether caveolae formation is involved in carbon nanoparticle-induced signaling processes, we quantified the formation of high molecular weight protein structures (>350 kDa) containing caveolin-1 under different exposure conditions (see Figure 2). In semi-quantitative Western-Blot analyses, we were able to demonstrate that treatment of the cells with carbon nanoparticles as well as with C6 ceramide led to an increase in high molecular weight caveolin-1 protein complexes while the treatment with EGF failed to induce this reaction (see Figure 2A). Increasing the antioxidant capacity of the cells by applying *N*-acetylcysteine as well as by adding the membrane-coupled antioxidant α-tocopherol, both reduced the amount of caveolin-1 protein complexes significantly (see Figure 2B,C). As expected, filipin III as an inhibitor of caveolae formation prevented the carbon nanoparticle-induced caveolin-1 protein complexes (see Figure 2D). These data strongly suggest that internalization of EGFR after carbon nanoparticle exposure of lung epithelial cells depends on the formation of caveolae.

### 2.4. Non-Canonical EGFR Activation In Vivo

**Figure 3:**
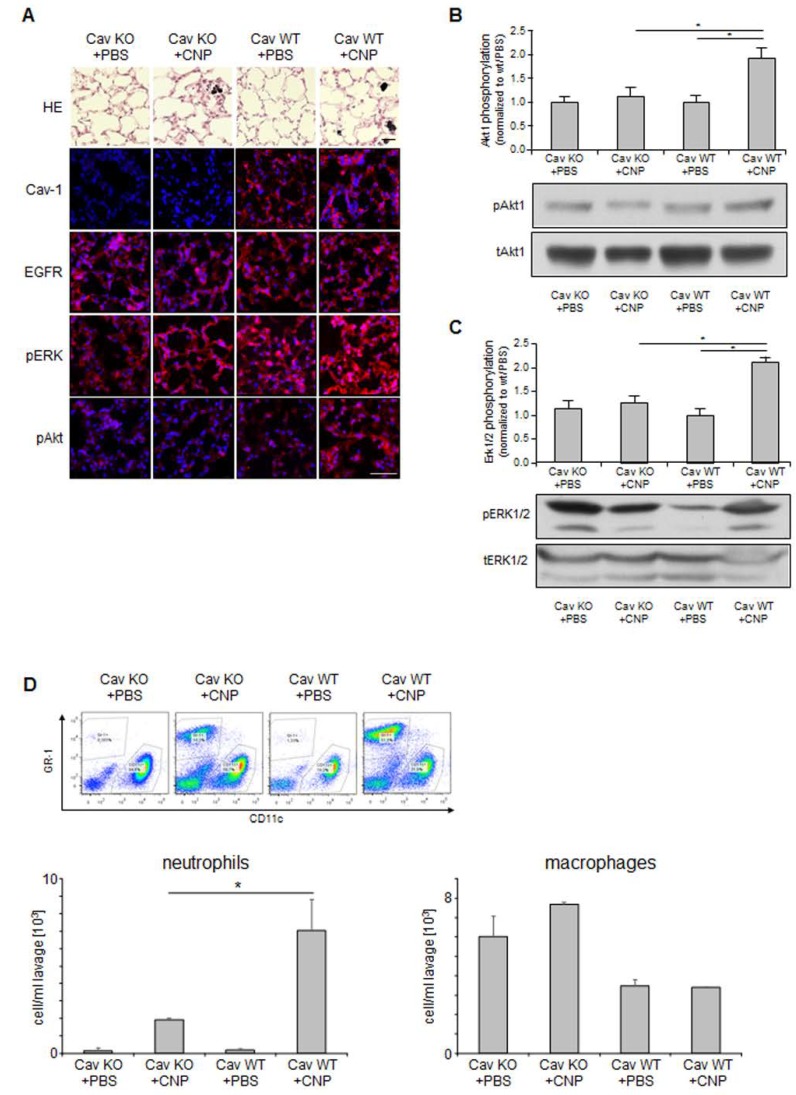
Non-canonical EGFR signaling in vivo. Caveolin-1 knock-out mice and their wild type littermates were exposed to carbon nanoparticles (CNP, 2.5 mg/kg) (or saline as control) by pharyngeal aspiration. Six hours after this single exposure, animals were sacrificed and subjected to bronchoalveolar lavage (BAL) followed by lung tissue preparation. (**A**) Immunohistochemistry from frozen sections of lung tissue. Lungs were either stained with Hematoxilin/Eosin (HE) or immunostained (red) for caveolin-1 (Cav-1), EGFR, phosphorylated Erk1/2 (pERK), or phosphorylated Akt (pAkt) and counterstained with DAPI (blue); (**B**) Relative phosphorylation of Akt in lung homogenates of animals were exposed as indicated. Means and standard errors of immune signals of phosphorylated Akt relative to total Akt and representative Western-blots; (**C**) Relative phosphorylation of Erk1/2 in lung homogenates of animals was exposed as indicated. Means and standard errors of immune signals of phosphorylated Erk1/2 relative to total Erk1/2 and representative Western-blots; (**D**) Flow cytometric analyses of lung lavages with respect to inflammatory cells. GR-1 positive cells (neutrophils) and CD11c positive cells (macrophages) were quantified per mL lung lavage. Scale bars represent 50 µm. *, significantly different from untreated control (*p* < 0.05).

The activation of MAPK signaling pathways via EGFR was identified as a specific mechanism by which carbon nanoparticles induce endpoints like proliferation, apoptosis, and pro-inflammatory responses in lung epithelial cells [3,28]. The in vivo relevance of this cellular reaction was earlier demonstrated in the lungs of animals exposed to carbon nanoparticles [29]. Investigations of signaling events after particle exposure demonstrated that the activation of the MAPK Erk1/2 and protein kinase B (Akt) are mediated by EGFR activation [11]. These EGFR-specific signaling pathways allowed us to test the relevance of caveolin-1-dependent EGFR activation in vivo. The specific appearance of phosphorylated forms of Erk1/2 and Akt was used as an indicator of non-canonical EGFR activation in vivo. The application of carbon nanoparticles in the lungs of animals is a well-established experimental system in which the specific interaction of nanoparticles with the airway epithelium can be investigated. Signaling events triggered by the particles as well as physiological reactions can be studied in tissue samples. The use of animals that lack the caveolin-1 gene due to genetic modification tested the relevance of non-canonical EGFR activation via caveolin-1 in vivo [30]. We, therefore, employed the system of pharyngeal aspiration of carbon nanoparticles (2.5 mg/kg) in the lungs of caveolin-1 knock out mice and their wild type littermates. The induction of signaling events was investigated 6 h after exposure (see Figure 3). At this time point after exposure, signaling events in lung epithelial cells are activated while inflammatory responses are still not at the peak. As described earlier, lungs of caveolin-1 deficient animals show morphological changes [30]. In hematoxilin eosin (HE) stained lung sections of these animals, we observed a mild phenotype of slightly thickened septa (see Figure 3A). Lung sections immuno-stained for caveolin-1 clearly show that this protein is present in wild type littermates. EGFR as well as the phosphorylated forms of Akt an Erk1/2 were detectable in lung epithelial cells. Exposure of WT-animals appeared to increase the activating phosphorylation of Akt and Erk1/2 while this reaction was not observed in exposed knock out animals. In order to verify this finding, the amount of phosphorylated signaling proteins was determined in protein preparations from lung homogenates. In these semi-quantitative analyses, the levels of phosphorylated Akt (see Figure 3B) and phosphorylated Erk1/2 (see Figure 3C) in relation to the respective amounts of total protein was elevated only in exposed WT animals. In animals lacking caveolin-1, both proteins were not activated after exposure to carbon nanoparticles.

The lack of caveolin-dependent activation of the Akt Erk1/2 signaling cascade is also obvious at the level of the inflammatory response in the lungs triggered by the nanoparticles. Neutrophilic granulocytes and macrophages as the major inflammatory cells were determined in lung lavages of exposed mice (see Figure 3D). In earlier studies, we were able to demonstrate that membrane-dependent signaling in lung epithelial cells is a major driver of neutrophil recruitment in the lung after particle exposure [14]. Accordingly, we now observed that the impairment of non-canonical EGFR activation in caveolin-1 deficient mice led to a marked reduction of this pro-inflammatory response. At the level of macrophages, such effects are not observed at this early time point after exposure. Caveolin-1 knock-out mice appear to have elevated macrophage numbers. This phenomenon appears not to influence the inflammatory response on nanoparticles. The results of the animal experiments demonstrate that carbon nanoparticles are able to address non-canonical EGFR activation via caveolin-1 in epithelial cells in vivo. However, the relevance of these signaling events in lung epithelial cells for nanoparticle-induced lung inflammation as a possible health effect of inhaled nanoparticles in humans is documented.

## 3. Discussion

The presented data demonstrate that EGFR is an important regulator of cellular functions and tissue homeostasis, which can be activated by carbon nanoparticles in epithelial cells via a non-canonical mechanism. This depends on caveolin-1. The induction of higher order structures built by caveolin-1 after carbon nanoparticle exposure and the suppressive effect of the inhibitor filipin III indicate that the formation of caveolae is a critical step involved in these cellular reactions. Our earlier studies identified Akt as a key signaling enzyme downstream of EGFR, which is also responsible for activating MAP-kinases Erk1/2 after nanoparticle exposure [11]. The in vivo experiments demonstrate that this specific signaling cascade, which is responsible for regulating epithelial tissue homeostasis and pro-inflammatory reactions in lungs exposed to nanoparticles depends on the structural protein caveolin-1. Furthermore, the inhibitor experiments demonstrate that signaling events upstream of EGFR activation include the generation of reactive oxygen species and the accumulation of ceramides in lipid raft membrane fractions, which we earlier identified as specific for the interaction of carbon nanoparticles with lung epithelial cells, are causative for the non-canonical activation of the EGFR.

Carbon nanoparticles can be considered model particles for combustion-derived environmental nanoparticles [31]. The inhalation of these particles has been linked to many pathological endpoints including neutrophilic lung inflammation and chronic obstructive pulmonary disease (COPD). Recent investigations have shown that lung epithelial cells are the most relevant cell type for the induction of neutrophilic lung inflammation triggered by carbon nanoparticles [32]. As the current data clearly demonstrate the link between non-canonical EGFR activation in this cell type and neutrophilic lung inflammation, the induction of this pathway may be considered as a measure for the toxicity of inhaled particles.

Besides toxicologically relevant incidents, carbon nanoparticles as well as other poorly soluble particles might be intentionally applied to the human body. There are a number of strategies that aim to employ carbon-based nanomaterials for diagnostic and therapeutic approaches in humans [33,34,35]. To our knowledge, a possible interference of these strategies with EGFR signaling is usually not tested. However, nanoparticle-based approaches that aim to suppress EGFR activity, like in tumor therapy, might have the side effect of an activation of this receptor pathway. Our in vivo findings indicate that these nanoparticle-specific effects can occur in vivo and can lead to physiological responses. Yet, there are applications in which the activation of membrane receptor kinases is wanted such as in regenerative therapy [36]. Recent developments of drug delivery systems aim to use nanoparticles for the application of growth factors among therapeutic targets [37]. By choosing appropriate carrier nanoparticles, non-canonical activation of EGFR and possibly other membrane receptor kinases could increase the effectivity of such therapeutic approaches.

## 4. Materials and Methods

### 4.1. Reagents

Carbon nanoparticles (CNP Printex 90, Degussa, Essen, Germany) and carbon particles (H. Haeffner, Chepstow, UK) were used for exposure experiments suspended in phosphate buffered saline (PBS). Particle characteristics as well as characteristics of the suspensions were determined as described earlier [19]. Physicochemical characteristics as well as methods of characterization are provided in the supplementary file.

### 4.2. Cell Culture and Exposure

RLE-6TN cells (ATCC, Manassas, VA, USA) were cultured as described earlier [3]. Cells grown to a confluence of 70–80% were used for exposure experiments. In order to discriminate exposure effects from serum-induced reactions, cells were kept at low serum conditions (0.5% fetal calf serum) for 20 h. Immediate early reactions of particle cell interaction were monitored five minutes after exposure to particle concentrations of 10 µg/cm^2^, which proved to be a relevant exposure dose that does not induce cytotoxicity, according to our earlier studies [3].

Inhibitors were added to the cells at 18 h (NAC (1 mM)), 4 h (ectoine (1 mM)), or 60 min with alpha-tocopherol (75 Μm), PP2 (10 μM), and filipin III (1 µg/mL) [38] prior to treatment with CNP (10 μg/cm^2^), CP (10 μg/cm^2^), C6-ceramide (5 μM), H_2_O_2_ (50 µM), or EGF (100 ng/mL). EGF (R&D Systems, Abingdon, UK) and ectoine ((*S*)-2-methyl-1,4,5,6-tetrahydropyrimidine-4-carboxylic acid, LPS-free, ultrapure 99%, bitop AG, Witten, Germany) were solubilized in sterile PBS. Filipin III (from *Streptomyces filipinensis*, Sigma-Aldrich Chemie, Schnelldorf, Germany), PP2 (Calbiochem, Schwalbach, Germany), and DSP (dithiobis-succimidylpropionate, Thermo Scientific, Waltham, MA, USA) were solubilized in DMSO (dimethyl sulfoxide) and diluted in PBS to the indicated concentrations. α-tocopherol (d-alpha-tocopherol succinate, semi-synthetic, Sigma-Aldrich Chemie, Schnelldorf, Germany) was solubilized in ethanol and further diluted in PBS before use. In experiments using these compounds, respective vehicle controls were performed. The effect of DMSO on lipid raft composition was investigated as described before [9]. DMSO treated samples showed no difference to PBS treated samples.

### 4.3. Protein Isolation

The cells were lysed on ice in modified radio immunoprecipitation assay buffer (25 mM Tris-Cl pH 7.4, 150 mM NaCl, 0.1 mM EDTA, 1% Nonidet P-40, 0.1% SDS, 1% deoxycholate, 0.025% NaN_3_, 1% protease inhibitor cocktail, 1% phosphatase inhibitor cocktail (both inhibitor cocktails from Sigma)), which was described in Reference [39]. Protein crosslinking by dithiobis-succimidylpropionate (DSP) was performed as described [27]. Cells were rapidly cooled to 4 °C prior to 1 h incubation with DSP (1 mM) at 4 °C. Crosslinking was stopped by adding 1 M Tris/HCl pH 7.4 (15 mM) prior to protein preparation. Afterwards, proteins were isolated as described above. Detergent resistant membrane raft fractions were isolated and detected as described earlier in Reference [9]. Cells were mechanically disrupted and treated with Triton X-100 (4-(1,1,3,3-tetramethylbutyl)phenyl-polyethylene glycol, 1%). Raft and non-raft fractions were collected from density gradients after ultracentrifugation. Raft fractions were identified by the presence of the raft marker ganglioside GM1 in dot blot assays, as described [9].

### 4.4. Protein Analyses

Western blotting was performed as described earlier [9]. Equal amounts of total cell protein (5–40 μg) were separated by using SDS-PAGE (7.5% or 10%) and transferred onto PVDF membranes (Hybond-P, Amersham Biosciences, Little Chalfont, UK). DSP-cross-linked caveolin-1 protein complexes were separated on 5% PAGE gels [26]. Unless otherwise stated, all antibodies were from Cell Signaling Technology (Danvers, MA, USA). The antibodies used include caveolin-1 (Upstate Biotechnology, Lake Placid, NY, USA), phospho-EGFR (Tyr^845^), Akt, phospho-Akt (Ser^473^), p44/42 MAPK, phospho-p44/42 MAPK (Thr^202^/Tyr^204^), and GAPDH (Imgenex Corp., San Diego, CA, USA). Signal strength was detected using the ECL Plus Western Blotting Detection System (Bio-Rad, Hercules, CA, USA). Band intensities from X-ray films (immune signal) were used for statistical calculations. The depicted graphs show either absolute immune signals (high molecular caveolin-1 complexes, EGFR pTyr^845^) or signals relative to the respective total proteins (Erk1/2, Akt).

### 4.5. Immunostaining

Cells were treated with 4% paraformaldehyde (20 minutes, room temperature). Permeabilisation and blocking was achieved by incubation with 3% bovine serum albumin and 0.3% Triton X-100 in PBS. Slides were incubated with primary antibodies (1:50) overnight at 4 °C, Akt, phospho-Akt (Ser^473^), Erk1/2, phospho-p44/42 MAPK (Thr^202^/Tyr^204^) (Imgenex Corp., San Diego, CA, USA). After 1 h of incubation with secondary antibodies (Alexa Fluor 594 or Alexa Fluor 488, 1:800 or 1:500; Invitrogen, Darmstadt, Germany), nuclei were counterstained by mounting with prolonged gold anti-fade mounting medium with DAPI (1:2000, Invitrogen). Cells were visualized using an Axiovert 200M microscope using (Zeiss, Jena, Germany, 400-fold enlargement, under oil). As control for the specificity of the reactions, mock immunostainings without primary antibodies were performed.

### 4.6. Animal Experiments

All animal experiments were approved by the local authorities in accordance with the German animal welfare legislation. Caveolin-1 knock-out mice [30] were generated by the group of T. Kurzchalia (Dresden, Germany). Knock-out and wild type mice were obtained by mating of heterozygous animals. Littermates either homozygous knock-out or wild type were used. Adult animals of both sexes were exposed as described earlier [19] by single pharyngeal aspiration of particle solutions (*n* = 4) or PBS (*n* = 3). Animals were sacrificed six hours after exposure. Broncho alveolar lavage was prepared from each lung. Lung tissue was sampled for histopathology. Lung sections (4–6 µm) were made from cryo-preserved lung tissue. Immunostainings were performed with the respective antibodies and fluorescent secondary antibodies, which were followed by embedding the sections in mounting medium that contains DAPI. Parallel sections were stained with hematoxylin/eosin. Immunostainings were analyzed microscopically. As a control for the specificity of the reactions, mock immunostainings without primary antibodies were performed. For semi-quantitative analyses of signaling proteins, tissue samples from two independent animal experiments were used (PBS *n* = 4–5, CNP *n* = 7–8).

Broncho alveolar lavages were subjected to differential cell counting. Inflammatory cells were discriminated by flow cytometry by employing a FACScanto II Flow Cytometer (BD Bioscience, BD Bioscience, Franklyn Lake, NJ, USA). Data were analyzed using FlowJo 7.6.5 software. Fluorescently labelled CD11c (N418) and GR-1 (RB6-8C5) (both BioLegend San Diego, CA, USA) were used to monitor changes in the inflammatory status of the lungs, which are reflected by shifts in the percentages of macrophages and neutrophils.

### 4.7. Statistical Analyses

For statistical analyses, one-way ANOVA followed by Bonferroni post hoc testing was performed using IBM SPSS statistics 22 (IBM Corp., Armonk, NY, USA). Results from Western-blot analyses of phosphorylated proteins were tested for statistical significance with Mann-Whitney U test. The sample size of the animal experiment was determined by power calculation using G*Power 3.1.9.2. Unless not otherwise stated, all experiments were performed as three independent replicates. Differences were considered as significant when *p* < 0.05. Bar graphs show means ± SEM.

## Supplementary Materials

The following are available online at http://www.mdpi.com/2079-4991/8/4/267/s1, Figure S1: Transmission elecron micrograph of CP after drying a particle suspension (H_2_O) on a holey carbon film, Table S1: Physical characteristics of CNP suspension in PBS.
