# Supplementary material for: Non-Canonical Activation of the Epidermal Growth Factor Receptor by Carbon Nanoparticles
Source: Nanomaterials (Basel). 2018 Apr 23;8(4):267. doi: 10.3390/nano8040267 (PMC5923597; doi:10.3390/nano8040267)
Supplement: Supplementary file 1 [file nanomaterials-08-00267-s001.pdf]

**Supplementary Materials**

Non-canonical activation of the epidermal growth factor receptor by carbon nanoparticles

Daniel Stöckmann, Tim Spannbrucker, Niloofar Ale-Agha, Philipp Jakobs, Christine Goy, Nadine Dyballa-Rukes, Tamara Hornstein, Alexander Kümper, Annette Kraegeloh, Judith Haendeler, Klaus Unfried

Physical analysis of carbon particle suspensions (CP=carbon particles; CNP carbon nanoparticles)
Method: Suspensions of particles in PBS [1 mg/ml] were prepared by ultrasound desintegration. Suspensions were analyzed by transmission electron microscopy (JEM-2100 LaB6, JEOL, Tokyo, Japan) and dynamic light scattering (Zetasizer, Malvern Instruments, Malvern, UK). Analyses were performed in triplicate. Results of the analyses performed for CNP have been published earlier.(Kroker *et al.*, 2015)

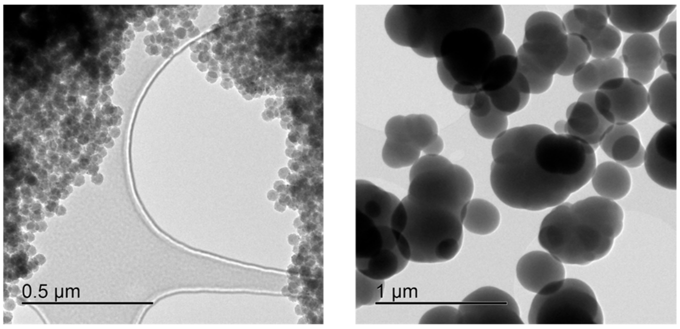


Figure S1.Transmission elecron micrograph of CP after drying a particle suspension ($H_2O$) on a holey carbon film.

Table S1: Physical characteristics of CNP suspension in PBS.

| | **zeta potential** [mV] | **mobility** [µmcm/Vs] | **conductivity** [mS/cm] | **size** [nm] peak1 [%] | **size** [nm] peak2 [%] | **size** [nm] peak3 [%] |
|---|---|---|---|---|---|---|
| CP | -16.0 | -1.257 | 15.63 | 359 (±22) 100 | - | - |
| CNP | -22.4 | -1.755 | 16.56 | 887 (±197) 88.8 | 118 (±126) 10.5 | 1798 (±3114.8) 0.7 |

Kroker M, Sydlik U, Autengruber A, Cavelius C, Weighardt H, et al. 2015. Preventing carbon nanoparticle-induced lung inflammation reduces antigen-specific sensitization and subsequent allergic reactions in a mouse model. Part Fibre Toxicol 12:20.
